# Introduction to the special issue: war and fun: exploring the plurality of experiences and emotional articulations of warfare and soldiering

**DOI:** 10.1080/07292473.2024.2409532

**Published:** 2024-10-01

**Authors:** Antonio De Lauri, Luigi Achilli, Iva Jelušić, Eva Johais, Heidi Mogstad

**Affiliations:** Chr. Michelsen Institute, Bergen, Norway

**Keywords:** war, fun, soldiers, emotions, moralities

## Abstract

In this text, we introduce the Special Issue ‘War and Fun: Exploring the Plurality of Experiences and Emotional Articulations of Warfare and Soldiering’ by highlighting the need to challenge, expand, and reorient public and scholarly debates in order to address the complex interplay of emotions, moralities and agency that characterise the human experience of war from the perspective of those who fight.

Passing between the companies that had been eating porridge and drinking vodka a quarter of an hour before, he saw everywhere the same rapid movement of soldiers forming ranks and getting their muskets ready, and on all their faces he recognized the same eagerness that filled his heart. ‘It [the war] has begun! Here it is, dreadful but enjoyable!’ was what the face of each soldier and each officer seemed to say. (Tolstoy, *War and Peace*)

Amid violence and gore, and despite – or sometimes because of – its shock and horror, war has always been marked by the presence of fun in manifold forms. From the Napoleonic wars and the trenches of the First World War to contemporary wars in Afghanistan, Ukraine, and Gaza, documented instances have revealed combatants and non-combatants engaging in actions diverging sharply from the grim expectations of warfare – collecting remnants of human bodies as trophies, creating videos or photos portraying enemies in humiliating postures (as we have seen extensively in the context of the USA’s War on Terror), intertwining sexual harassment with pranks within their ranks, toying with lingerie found in civilian homes (as Israeli soldiers did in Gaza), or engaging in seemingly innocuous activities such as playing Pokémon during military actions. As exemplified by the Tolstoy quotation above, films, music, memoirs, and novels likewise can represent war-fighting as an exciting and pleasurable undertaking.^1^

With some exceptions, scholars have often turned a blind eye to this element of warfare. Specifically, the complex and multifaceted relationship between fun and war has never been the focus of theorisation and empirical scrutiny in the social sciences. This special issue seeks to address this gap and thereby contribute to a more nuanced understanding of war rooted in the lived experiences of those who fight and endure war.

The five co-authors of this introduction are members of the interdisciplinary research project *War and Fun: Reconceptualising Warfare and Its Experience (WARFUN)* funded by the European Research Council.^2^ This special issue of *War & Society* contains individual articles written by project members plus additional contributions from historians, social anthropologists, and sociologists working in different historical and geographical contexts. Together, the ten articles represent a crucial step towards dispelling the ‘fog of war’ within academic discourse. In different ways, the contributions challenge, expand, and reorient public and scholarly debates, offering fresh insights into the complex interplay of emotions and agency that characterise the human experience of war.

The suffering and hardships that humans endure within and because of war cannot be stressed enough. Wars injure and destroy people’s lives and homes, social relations and infrastructure, and national and international projects and relations. In light of this, the focus on fun might occur to some readers as trivialising violence and human suffering. Admittedly, this special issue challenges normative epistemologies and dominant moralities of warfare but it is not the intention to downplay the hardship and devastation that war causes. Instead, fun here serves as an entry point to unveil the plurality of experiences and emotional articulations that are usually neglected by normative approaches. By exploring fun and related experiences and sensations such as thrill, humour, and pleasure, the contributions achieve a deeper understanding of the lived realities of war. The special issue demonstrates that it is crucial to examine the seemingly paradoxical relationship between war and fun to understand the continuous allure of war amid its violence and destruction.

The remainder of the introduction is divided into three sections. In the first section, we examine the dearth of scholarly theorisation of fun in war and consider some of the effects of this scholarly gap. Next, we elaborate on the conceptual framework that integrates fun in the study of war. The third and final section gives an overview of the themes and findings that the ten articles featured in this issue of *War & Society* offer.

## War and fun: the blind spot of war epistemologies

The study of fun in war presents a curious lacuna in social science research. On the one hand, war has received ample attention as a modality of power,^3^ a practice of sovereignty,^4^ a process of state-making,^5^ a project to exercise the right to kill,^6^ a way towards freedom,^7^ a force for good,^8^ and a battle over limited resources.^9^ On the other hand, sociologists, behavioural scientists, and psychologists have studied fun as a process of socialisation, a mechanism of relationality and identification, and a way to enable specific abilities or ritualise ordinary life.^10^ As both contemporary and historical accounts of war attest, these social and psychological functions also apply to groups and individuals involved in war-making, and the joy of camaraderie and the thrill of combat are integral elements of warrior culture. The two topics – war and fun – have however not been brought together. Since fun in war *is* an empirical phenomenon, this raises the question of why it has largely slipped scholarly attention.

The reasons are likely manifold and varied across cultural and institutional contexts and disciplines, yet we suggest that there might be some overarching explanations. Specifically, we propose that many studies of war and soldiering are driven and coloured by moral and normative understandings of war as a deeply tragic and regrettable event from the perspective of victims of war or external actors.^11^ Dominant studies and narratives of war, steeped in violence, grief, and suffering, align with the normative perceptions of war’s impact – its capacity to disrupt peace, inflict bodily harm, generate suffering, and leave enduring social and environmental scars. This focus is expected, given the profound and devastating consequences of conflict. Yet this perspective overshadows the fact that war, for those who experience it first-hand, might not represent a monolithic experience of horror and trauma. Instead, it can encompass a broader spectrum of human emotions, including laughter, joy, compassion, humour, excitement, friendship, love, and kindness (see also Tomforde, and Maringira this issue).^12^ The academic gap, thus, lies not only in the scarcity of studies focusing on this interplay but also in the limited acknowledgement of the multifaceted nature of war experiences, where the dire and the jubilant not only coexist but interweave, shaping the lived realities of those within the theatre of war.

The limited attention to fun in war has significant ramifications for academic research and our broader comprehension of conflict. Firstly, this omission has relegated emotions largely to the realm of the private, hindering a thorough grasp of how politics and emotions intertwine within the framework of war.^13^ Emotions play a crucial role in shaping the experiences and actions of combatants and non-combatants alike. By ignoring these aspects, we miss out on a comprehensive understanding of the multifaceted human experience of war. Secondly, the neglect has been shaped by, but has also reinforced, a culturally and historically conditioned ‘morality of war’ that works to discipline scholarly attitudes and analyses. To begin with, this morality of war frames war and fun as antithetical and the study of fun in war as trivial if not morally inappropriate and provocative. The prevalence of this attitude was brought home to us several times when seeking research access (see Johais, this issue)^14^ or presenting our project to colleagues and other scholars (see Mogstad, this issue).^15^

More problematically, the morality of war has also marginalised the presence of *different moralities* expressed by those directly involved in war. To be more specific, scholarly analyses have often focused on the ethical quandaries faced by soldiers and fighters, often labelled as ‘moral dissonance’^16^ or ‘moral conflict’. Increasingly, scholars have also paid attention to war-related stress arising from such moral conflicts, described as ‘moral injury’.^17^ While this research has played an important role in humanising soldiers, it has often assumed that soldiers’ moralities are based on deeply held and coherent beliefs, including biologically engrained resistance to violence.^18^ Arguably, the prevalent focus on soldiers’ moral conflicts and injuries has also curtailed more open-ended explorations of veterans’ dynamics of identity,^19^ as well as combatants’ broad plurality of moral attitudes, attachments, and convictions.

Thirdly, the lack of systematic studies of fun in war has allowed state and military institutions to treat undesirable and cruel incidents as exceptional excesses and deviant behaviour of individuals (see De Lauri, this issue). For example, when British soldiers beat Iraqi citizen Baha Mousa to death in 2003 during the second Iraq war, the prosecutors decided they could not consider it to be an act of torture since it was done ‘for fun’ rather than to collect information or to intimidate.^20^ Such a decision illustrates the implications of the widely accepted assumption that fun is alien to the way that war is conducted by soldiers on the ground.

To be sure, there are studies of soldiers which show that they enjoy morale-boosting activities, military drills, and entertainment offered at military bases, and also that they adapt them to their exigencies.^21^ Moreover, studies of soldierly memoirs and lifeworlds have highlighted their lust for combat and the presence of emotions such as ‘relief, elation and excitement’.^22^ Historical research has highlighted articulation of different soldiers’ responses to extreme situations.^23^ There is also a growing literature on joy and pleasure,^24^ as well as on jokes, laughter, and humour, in wartime.^25^ More broadly, recent studies have also started to address the complex array of feelings and sensations in war.^26^ Nonetheless, questions related to the moral, strategic, psychological, emotional, and social implications of fun in war remain surprisingly unexplored in the social sciences.

Moreover, as Mogstad argues in this issue, experiences of fun in war are usually analysed in functional terms as a coping mechanism or stress release. Alternatively, activities that are experienced as funny or pleasurable are commonly said to promote bonding and unit cohesion and therefore analysed as instrumental for soldiers’ well-being and war efforts. In this special issue, we critically examine the work practices of fun and humour do to sustain war ‘from below’. We also present other analyses that depart from functionalist readings and show how war can be construed as an ordinary or everyday experience (see Mogstad, this issue).

## WARFUN: the conceptual nexus

To reiterate, it is undeniable that war brings about immense pain and loss, often leaving indelible marks on the landscapes it touches and the lives it alters for generations. At the same time, ‘there is more to war than pain and suffering’.^27^ Building on internal and public discussions we have had in the WARFUN team, this special issue ventures into a domain often overlooked in public narratives and scholarship on war: the role of fun, and related ‘positive’ feelings such as humour, pleasure, and love, amid the violence and turmoil of conflict.

Recognising the need for an epistemological shift in how we learn about and discuss war, we explore the complexity of war participation beyond traditional frameworks. The goal of this special issue is to provide a comprehensive understanding of war and soldiering through the lens of fun and its related phenomena. By examining these aspects, it seeks to shed light on the varied experiences of those directly involved in conflicts, offering nuanced perspectives on the emotional and psychological dynamics at play. This endeavour not only contributes to filling a significant gap in the current research landscape but also challenges us to rethink our perceptions of war and soldiering, and the complex interplay of emotions that accompany them.

The concept of fun carries a wide range of connotations, from light-hearted joy to more sinister aspects.^28^ Nonetheless, the meaning and experience of fun are often taken for granted and left untheorised.^29^ Within the context of this special issue, fun serves as an entry point into a deeper realm of soldiering and warfare. The richness and heterogeneity of the contributions, spanning diverse temporal and geographical contexts, underscore the complexities of defining ‘fun’ in the milieu of war. We intentionally eschew offering a rigid definition of fun, recognising its evolution and multifaceted nature across different war scenarios. Common to all contributors though is a recognition of fun as an embodied, emotional, and social experience that is morally agnostic. Drawing on different qualitative research methods – including ethnographic interviews, fieldwork, and archival sources – the contributors offer theorisations of war building on the plurality of social actors’ experiences and emotions. We explore the capacity of fun, in its various manifestations, to redefine social categories and practices, its impact on the personal experiences of those involved in war, and the ways it can generate new forms of agency and possibilities for action.

This special issue’s contributors present interpretations and analyses that both complement and challenge each other. For instance, some look specifically at the work of humour (Hamer; Rastrilla and Donofrio) while others propose that fun is an emotional experience that is distinct from humour and seek to move beyond functionalist analyses (Mogstad). In other articles, fun is conceptualised as a form of communication and social practice that (directly and indirectly) engages publics, subverts norms, and at times supports the structures it appears to oppose. In the latter configuration, it serves as a ‘ritual of inversion’, challenging the formal objectives of war while simultaneously adhering to its overarching goals of dominion and control.^30^

This nuanced understanding of fun, grounded in empirical evidence and ethnographic richness, mirrors the complexity of war itself. It highlights how individuals involved in conflict recognise and experience fun, illustrating that these perceptions and experiences can vary significantly across different contexts and cultures. War is not merely a series of top-down political decisions but is also shaped by the myriad ways individuals engage with it from below.^31^ As war and its definitions have evolved over the centuries, so too have the ways people find meaning, cope with stress, dehumanise or rehumanise the enemy, or seek moments of levity and connection amid the chaos.

As we delve into the stories and studies presented in this special issue, we begin to see war not just through the lens of its political and strategic dimensions but also through the personal experiences and emotional landscapes of those who both perform and endure war. By focusing on various military actors, both combatants and non-combatants, we illuminate the nuanced ways in which individuals navigate the complex landscapes of war. This approach reveals the interplay between the overarching structures of military engagement and the personal, subjective agency of those within it. It demonstrates how individuals and groups within these settings utilise both tactical and spontaneous strategies of fun and leisure as essential components of their warfare practices. Experiences of fun, we show, are commonplace in war and take place both because of, and despite, its violence and horror. At the same time, experiences and representations of fun in war are always contingent and shaped by cultural, historical, and political factors and sensibilities.

## Fun in war: the emotional and experiential spectrum

The findings of our special issue delve into various dimensions of how fun intersects with the experiences of warfare, shedding light on its roles in shaping practices, narratives, and the very fabric of conflict environments.

### Making soldiers

Fun is a social phenomenon that plays an important role in creating collective identities,^32^ which are formed around a shared universe of experience, by negotiating norms of (in)appropriateness and through distinguishing insiders from outsiders.^33^ Just like in other social milieus, armed forces use the mode of play to socialise soldiers into their new roles and instil a military culture. Moreover, the experience of fun in war can be deeply transformative. Soldiers’ testimonies and memoirs show, for example, how people’s descent into the absurdity of war profoundly transforms them. This creates the possibility for different forms of subjectivity to emerge.^34^ This special issue thus tackles the ways in which having fun in war contributes to the formation of subjectivities and agency. Specifically, it adds to the literature that highlights the role of humour in the becoming of the soldier subject.^35^

Illustrating this transformative process, Luigi Achilli explores the role of ‘fun’ in child recruitment by the Boko Haram militia in Nigeria. The contribution posits that ‘fun’, along with associated positive emotions, influences children’s experiences, blurs the victim–perpetrator boundary, and contributes to group perpetuation. Likewise, Blasco Sciarrino posits that the conception of war as a game increased the combat effectiveness and violent tendencies of the Italian shock troops known as *Arditi* in the First World War. In this case, however, many aspiring soldiers already viewed war as a ludic event even before they joined the Arditi due to the political cultures and ideologies in which they believed. Yet recourses to fun and play and an intense sports-based drilling regimen further nurtured the ‘war as game’ mindset. By revealing how fun shapes ways of participation and complicity, these contributions ultimately highlight its facilitating role in the unfolding of wars.

### Normalisation of war

Prior research across the social sciences has underscored the human inclination to seek normality through the pursuit of joy and fun amid the harrowing realities of conflict.^36^ Building on these and other studies,^37^ this special issue explores the multifaceted role of fun in reconstructing social worlds amid and in the aftermath of violence. The exploration through the lens of fun shows how combatants and non-combatants endure wars and often find moments of joy, camaraderie, and meaning. In a variety of case studies, the contributors delve into the intricacies of fun as a coping mechanism and reveal that certain activities considered as fun, such as telling jokes, playing games, singing, and dancing, generate a greater degree of social integration and affirmation of common values. Addressing these issues is essential for countering instances of public discourse that tend to focus solely on the adversity and suffering caused by war or oversimplify the idea of soldiers as either killing machines or humanitarian saviours.^38^

Importantly, contributions also venture beyond recognising fun as a mechanism of social rebuilding. They critically examine the intricate process through which fun possesses the capacity to ‘undo’ the social fabric, challenging and transforming social norms, identities, and relationships. This dual perspective offers a nuanced understanding of fun’s power to both construct and deconstruct, highlighting its significance in the dynamic interplay between continuity and change in societies touched by war. Through their ethnographic richness, our contributors extend the debate, revealing the complex ways in which fun shapes, and is shaped by, the exigencies of conflict and the human struggle for resilience and meaning.

In a historical example, Laura Pérez Rastrilla and Andrea Donofrio analyse cartoons authored by Andrés Martínez de León during the Spanish Civil War (1936–39). The article uncovers the narrative and visual techniques that allowed audiences, whether they were combatants or non-combatants, to find enjoyment amid the war. Through content analysis of textual and graphic elements of the cartoons, the article reveals how humour served as a daily strategy of critical, cultural, and subversive resistance to pain and fear.

Similarly, Petra Hamer underscores humour’s role as a coping mechanism during the Bosnian War in besieged Sarajevo (1992–5). Hamer particularly focuses on dark humour expressed through ethnic jokes, characterised by irony, sarcasm, and satire. Despite being primarily intended for entertainment, Hamer argues, these jokes reveal underlying tensions between groups involved in the war. Fuelled by various nationalisms, humour and having fun could be used to determine who belonged, and who did not. Through analysing these forms of humour, the article provides insight into how laughter intertwined with the complexities of war, identity, and nationalism.

In turn, Iva Jelušić examines the experiences of participants in the Second World War battles of Neretva and Sutjeska, specifically members of the Theatre of the People’s Liberation. Her contribution explores how organised entertainment functioned amid the two most difficult battles fought on the territory of Yugoslavia, and what kind of fun soldiers favoured. At the same time, the study sheds light on war participants’ experiences of togetherness, sharing common goals, and spite towards the enemy.

Contributors also examine soldiers’ lust for battle and romance in the war zone and how they experience and process these sensations during and in the aftermath of war. Maren Tomforde examines soldiers’ experiences of joy and battle lust as elements on a continuum of emotions that occur alongside fear, doubt, and moral conflict. Drawing on the accounts of German elite soldiers who served in Afghanistan, she offers a nuanced understanding of how soldiers process their combat experiences and use of violence.

Whereas the soldierly profession is characterised by the application of violence, Godfrey Maringira draws attention to soldiers’ humaneness and the continuities between soldier and civilian life. In the life stories of former Zimbabwean soldiers, war appears as a space of social mutuality. By establishing relationships with local civilians in the war zone, soldiers gained an understanding of the environment in which they were deployed, shaped the war context, and could define it as home.

While pushing in different directions, these studies of cultural production and artistic performance, human emotions, and relationships in war zones all enhance our knowledge of everyday life under the condition of war. Although war disrupts the social order, the contributions show that war experiences are rarely as exceptional as expected but continue civilian social practices and create a ‘new normal’. Taken together, they bespeak the reproductive effect of fun for war as a social institution – fun makes it possible to live through and endure it.

### Soldiers’ and society’s war morality

Finally, the special issue discusses how fun is often considered antithetical to war and that suggesting otherwise can provoke moral indignation and resistance from society and researchers alike. To be sure, not all forms and expressions of fun in war are considered morally problematic or unintelligible. As several of the contributions in the special issue show, the military uses fun for recruitment, play during training, and entertainment to dispel sorrows and fill free time. Hence, the military considers fun in war as acceptable and useful when it fulfils desirable functions such as building soldierly cohesion or stress release, which likely improve military performance. Likewise, civilians – including military critics and researchers – often recognise soldiers’ emotional or psychological need to have fun to cope with what they have witnessed and experienced in warfare.

Experiences and representations of fun that exceed these functions and boundaries, however, are frequently viewed with surprise or disapproval. As this special issue shows, this is particularly the case in some national and military cultures due to their respective histories and self-understandings. Illustrating this, Eva Johais conceptualises the indignant reactions to the suggestion of a WARFUN nexus as an outright taboo which she explains in the case of Germany with the country’s antimilitarist political culture. She further shows that German soldiers found it problematic to manage the discrepancy between civil and military culture. On the one hand, German soldiers are expected to behave as ‘citizens in uniform’ and have internalised the WARFUN taboo themselves. But on the other hand, they experienced that humour forms an integral part of soldier culture.

In contrast, the Norwegian soldiers Heidi Mogstad studies welcomed the difference between civil and military life as they found in Afghanistan precisely the thrill and adventure that their everyday life and work at home lacked. They also linked their experiences of fun to mastery, and specifically the mastery of combat and other ‘risky missions’. The idea that Norwegian soldiers were having fun in Afghanistan, and especially having fun while engaging in combat, is difficult to digest for a Norwegian public used to thinking of their soldiers in heroic and humanitarian terms. Like the German soldiers in Johais’s study, the Norwegian soldiers thus refrained from representing their service in Afghanistan as fun when interacting with family members and civilians they knew would have difficulties understanding. Seeking to understand the Norwegian soldiers’ experiences ethnographically, Mogstad argues that their sensations of fun in Afghanistan were shaped by their mode of soldiering, specifically their asymmetric and distant relationship to the Afghans they were tasked to fight or protect. Moving beyond normative and functionalist approaches, she further highlights the importance of examining soldiers’ moralities, including their sense of responsibility and innocence.

In conclusion, this special issue not only bridges a significant gap in scholarly discourse but also aims to initiate a broader conversation about the complexities of warfare beyond conventional narratives of violence and tragedy. The process of compiling these studies has itself been a testament to the complexity and sensitivity of the topic. The WARFUN team frequently encountered emotional reactions and resistance when presenting our findings to friends, colleagues, and academic communities. These reactions underscored the prevailing discomfort and societal taboos surrounding the juxtaposition of war and fun, revealing a deeply ingrained reluctance to associate war with anything other than suffering and loss. This resistance often mirrors the moral dilemmas that the project aims to explore, highlighting the need for continued dialogue and reflection on how war is perceived and discussed in both public and academic spheres. At the same time, we would like to emphasise that veterans, soldiers, and fighters have presented us with very different reactions, being immediately able to relate to the emotional and moral complexities that active participation in war implies (see De Lauri, this issue).

We are profoundly grateful to *War & Society* for providing a platform for this vital discourse and anticipate that future research will further illuminate the intricate interplay of emotions in war, contributing to a deeper and more nuanced comprehension of soldiering and warfare. By embracing this broader emotional and experiential spectrum, we can better appreciate the human condition in times of conflict, and two aspects in particular: the diverse ways in which individuals find resilience, meaning, and even moments of joy in the direst of circumstances; and the way acts of inhumanity, cruelty, and terror are normalised, reproduced, or explained in war contexts.

## Footnotes

1 Synne L. Dyvik, ‘“Valhalla Rising”: Gender, Embodiment and Experience in Military Memoirs’, *Security Dialogue* 47, no. 2 (2016), 133–50; Chris Hedges, *War Is a Force that Gives Us Meaning* (New York: Anchor Books, 2023); William Broyles Jr, ‘Why Men Love War’, *Esquire* (1984), <https://www.esquire.com/news-politics/news/a28718/why-men-love-war/> [accessed April 29, 2024].

2 WARFUN is a European Research Council (ERC) Consolidator Grant project (Grant agreement 101001106) led by Antonio De Lauri at the Chr. Michelsen Institute, <https://www.cmi.no/projects/2535-erc-war-and-fun>.

3 Paul Q. Hirst, *War and Power in the 21st Century* (New York: Polity, 2001).

4 Michael Green, ‘War, Innocence, and Theories of Sovereignty’, *Social Theory and Practice* 18, no. 1 (1992), 39–62.

5 Charles Tilly, ‘War Making and State Making as Organized Crime’, in *Bringing the State Back In*, edited by P. Evans, D. Rueschemeyer, and T. Skocpol (Cambridge: Cambridge University Press, 1985).

6 Achille Mbembe, ‘Necropolitics’, *Raisons Politiques* 21, no. 1 (2006), 29–60.

7 Antonio De Lauri, ‘The Taliban and the Humanitarian Soldier: Configurations of Freedom and Humanity in Afghanistan’, *Anuac* 8, no. 1 (2019), 31–57.

8 Claire Duncanson, *Forces for Good? Military Masculinities and Peacebuilding in Afghanistan and Iraq* (Houndmills: Palgrave Macmillan, 2013).

9 Philippe Le Billon, ‘The Political Ecology of War: Natural Resources and Armed Conflicts’, *Political Geography* 20, no. 5 (2001), 561–84.

10 Erving Goffman, *Encounters: Two Studies in Sociology of Interaction* (Indianapolis: The Bobbs-Merrill Company, 1961); Walter Podilchak, ‘The Social Organization of Fun’, *Leisure and Society* 8, no. 2 (1985), 685–92; idem, ‘Establishing the Fun in Leisure’, *Leisure Sciences* 13, no. 2 (1991), 123–36; Gary Alan Fine and Ugo Corte, ‘Group Pleasures: Collaborative Commitments, Shared Narrative, and the Sociology of Fun’, *Sociological Theory* 35, no. 1 (2017), 64–86.

11 Antonio De Lauri, ‘Introduction’, *Antropologia* 16 (2013), 723; idem, ‘The Experience of War: The WARFUN Project’, *European Dissemination – Project Repository Journal* 14 (2022), 90–93; Thomas R. Pedersen, ‘Breaking Bad? Down and Dirty with Military Anthropology’, *Ethnos* 86, no. 4 (2021), 676–93.

12 Antonio De Lauri, ‘The Idea of a Clean and Efficient War Is a Dangerous Lie’, *Common Dreams* (2022), <https://www.commondreams.org/views/2022/04/08/idea-clean-and-efficient-war-dangerous-lie> [accessed April 26, 2024]; Maren Tomforde, ‘Processing Violence: The Continuum between Fear, Doubt, and Joy among German Soldiers in Afghanistan’, *War & Society* 44, no. 1 (2025); Godfrey Maringira, ‘Wartime Soldiers, Civilian Relations: Zimbabwean Soldiers in the Democratic Republic of Congo War 1998–2002’, *War & Society* 44, no. 1 (2025).

13 Linda Åhäll and Thomas Gregory, eds., *Emotions, Politics and War* (Abingdon: Routledge, 2015).

14 Eva Johais, ‘The WARFUN taboo’, *War & Society* 44, no. 1 (2025).

15 Heidi Mogstad, ‘“Playing war”: Norwegian Soldiers’ Experiences of Fun and Responsibility in Afghanistan’, *War & Society* 44, no. 1 (2025).

16 Peter French, *War and Moral Dissonance* (Cambridge: Cambridge University Press, 2010).

17 Robert Emmet Meagher and Douglas A. Prye, eds., *War and Moral Injury: A Reader* (Eugene, OR: Cascade Books, 2018); Jonathan Shay, *Achilles in Vietnam: Combat Trauma and the Undoing of Character* (New York: Atheneum, 1994).

18 Kenneth MacLeish, ‘How to Feel about War: On Soldier Psyches, Military Biopolitics, and American Empire’, *BioSocieties* 14, no. 2 (2019), 274–99; Tine Molendijk, *Moral Injury and Soldiers in Conflict: Political Practices and Public Perceptions* (Abingdon: Routledge, 2021).

19 Jeni Hunniecutt, *Rethinking Reintegration and Veteran Identity: A New Consciousness* (New York: Palgrave Macmillan, 2022).

20 Tobias Kelly, ‘Why are “others” always guilty of torture?’, Opinion, 8 November 2011, Al Jazeera: <https://www.aljazeera.com/opinions/2011/11/8/why-are-others-always-guilty-of-torture> [Accessed July 1, 2024.].

21 Antonio De Lauri, ed., *WARFUN Diaries*, Vol. 1 and Vol. 2 (Bergen: CMI and ERC, 2022, 2023); Clémentine Tholas-Disset and Karen A. Ritzenhoff, eds., *Humor, Entertainment, and Popular Culture during World War I* (New York: Springer, 2015); Jason Crouthamel, ‘Cross-dressing for the Fatherland: Sexual Humour, Masculinity and German Soldiers in the First World War,’ *First World War Studies* 2, no. 2 (2011), 195–215.

22 Dyvik, 143; Thomas R. Pedersen, ‘Get Real: Chasing Danish Warrior Dreams in the Afghan “Sandbox”‘, *Critical Military Studies* 3, no. 1 (2017), 7–26, at 12.

23 Graham Seal, ‘“We’re Here Because We’re Here”: Trench Culture of the Great War’, *Folklore* 124, no. 2 (2013), 178–99; Elissa Mailänder, ‘Making Sense of a Rape Photograph: Sexual Violence as Social Performance on the Eastern Front, 1939–1944’, *Journal of the History of Sexuality* 26, no. 3 (2017), 489–520.

24 Julia Welland, ‘Joy and War: Reading Pleasure in Wartime Experiences’, *Review of International Studies* 44, no. 3 (2018), 438–55; Jean-Yves Le Naour, ‘Laughter and Tears in the Great War: The Need for Laughter/the Guilt of Humour’, *Journal of European Studies* 31 (2001), 265–75.

25 Katherine E. Brown and Elina Penttinen, ‘“A Sucking Chest Wound Is Nature’s Way of Telling You to Slow Down …”: Humour and Laughter in War Time’, *Critical Studies on Security* 1, no. 1 (2013), 124–26; Martina Kessel, ‘Laughing about Death? “German Humor” in the Two World Wars’, in *Between Mass Death and Individual Loss: The Place of Death in Twentieth-Century Germany*, ed. by Alon Confino, Paul Betts, and Dirk Schumann (Oxford: Berghahn Books, 2008).

26 Victoria Basham, ‘Waiting for War: Soldiering, Temporality and the Gendered Politics of Boredom and Joy in Military Spaces’, in *Emotions, Politics and War*, ed. by L. Åhäll and T. Gregory (Abingdon: Routledge, 2015); Brooke Johnson, ‘The Erotic as Resistance: Queer Resistance at a Militarized Charter School’, *Critical Military Studies* 5, no. 3 (2019), 195–212; Hanne Watkins and Brock Bastian, ‘Lest We Forget: The Effect of War Commemorations on Regret, Positive Moral Emotions, and Support for War’, *Social Psychological and Personality Science* 10, no. 8 (2019), 1084–91.

27 Elina Pentinnen, *Joy and International Relations: A New Methodology* (Abingdon: Routledge, 2014), 4.

28 John Kerbs and Jennifer Jolley, ‘The Joy of Violence’, *American Journal of Criminal Justice* 32, no. 1–2 (2007), 12–29; John Parry, ‘Just for Fun: Understanding Torture and Understanding Abu Ghraib’, *Journal of National Security Law and Policy* 1 (2005), 253–75; Podilchak, ‘Social Organization of Fun’; idem, ‘Distinctions of Fun, Enjoyment and Leisure’, *Leisure Studies* 10, no. 2 (1991), 133–48; Gary Alan Fine and Ugo Corte, ‘Dark Fun: The Cruelties of Hedonic Communities’, *Sociological Forum* 37, no. 1 (2022), 70–90.

29 Ben Fincham, *The Sociology of Fun* (New York: Palgrave Macmillan, 2016).

30 De Lauri, ‘Experience of War’.

31 De Lauri, ‘Introduction’.

32 Fincham, 19.

33 Eyal Ben-Ari and Liora Sion, ‘“Hungry, Weary and Horny”: Joking and Jesting among Israel’s Combat Reserves’, *Israel Affairs* 11, no. 4 (2005), 669; Gary A. Fine and Michaela de Soucey, ‘Joking Cultures: Humor Themes as Social Regulation in Group Life’, *HUMOR* 18, no. 1 (2005), 1 − 22.

34 Oskar Verkaaik, *Migrants and Militants* (Princeton: Princeton University Press, 2004).

35 Richard Godfrey, ‘Soldiering On: Exploring the Role of Humour as a Disciplinary Technology in the Military’, *Organization* 23, no. 2 (2016), 164 − 83; Beate Sløk-Andersen, ‘The Butt of the Joke? Laughter and Potency in the Becoming of Good Soldiers’, *Cultural Analysis* 17, no. 1 (2019), 25 − 56; Joanna Tidy, ‘The Part Humour Plays in the Production of Military Violence’, *Global Society* 35, no. 1 (2021), 134 − 48.

36 Ivana Maček, *Sarajevo under Siege: Anthropology in Wartime* (Philadelphia: University of Pennsylvania Press, 2009); Roxani Krystalli and Philipp Schulz, ‘Taking Love and Care Seriously: An Emergent Research Agenda for Remaking Worlds in the Wake of Violence’, *International Studies Review* 24, no. 1 (2022), 1–25.

37 Veena Das et al., *Remaking a World: Violence, Social Suffering, and Recovery* (Berkeley: University of California Press, 2001).

38 Antonio De Lauri, ‘Humanitarian Militarism and the Production of Humanity’, *Social Anthropology* 27, no. 1 (2019), 84–99; Heidi Mogstad, ‘The Self-Realising Soldier: Post-Heroic Reflections from Norwegian Afghanistan Veterans’, *Public Anthropologist* 6 (2024), 1–44.

